# Genetic Polymorphisms and In Silico Mutagenesis Analyses of *CYP2C9*, *CYP2D6*, and *CYPOR* Genes in the Pakistani Population

**DOI:** 10.3390/genes9100514

**Published:** 2018-10-22

**Authors:** Shabbir Ahmed, Jie Zhou, Zhan Zhou, Shu-Qing Chen

**Affiliations:** 1 Institute of Drug Metabolism and Pharmaceutical Analysis and Zhejiang Provincial Key Laboratory of Anti-Cancer Drug Research, College of Pharmaceutical Sciences, Zhejiang University, Hangzhou 310058, China; shabbirpharma@gmail.com (S.A.); zhoujie127@zju.edu.cn (J.Z.); 2 International Center for Precision Medicine, Zhejiang California International NanoSystems Institute (ZCNI), Hangzhou 310058, China

**Keywords:** pharmacogenomics, drug-metabolizing enzymes, polymorphism, alleles frequencies, in silico mutagenesis, Pakistani population

## Abstract

Diverse distributions of pharmacogenetically relevant variants of highly polymorphic *CYP2C9*, *CYP2D6* and *CYPOR* genes are responsible for some varied drug responses observed across human populations. There is limited data available regarding the pharmacogenetic polymorphisms and frequency distributions of major allele variants in the Pakistani population. The present in silico mutagenesis study conducted on genotype pharmacogenetic variants and comparative analysis with a global population aims to extend the currently limited pharmacogenetic available evidence for the indigenous Pakistani population. Extracted genomic DNA from 244 healthy individuals’ venous blood samples were amplified for distinct variant loci in the *CYP2C9*, *CYP2D6* and *CYPOR* genes. Two-way sequencing results were compared with standard PubMed data and sequence variant loci confirmed by Chromas. This study revealed significant variations in *CYP2C9* (rs1799853, rs1057910 and rs72558189), *CYP2D6* (rs16947 and rs1135840), and *CYPOR* (rs1057868, rs781919285 and rs562750402) variants in intraethnic and interethnic frequency distributions. In silico mutagenesis and three-dimensional protein structural alignment analysis approaches clearly exposed the possible varied impact of rare *CYPOR* (rs781919285 and rs562750402) single nucleotide polymorphisms (SNPs) and confirmed that the influences of *CYP2C9* and *CYP2D6* variants are consistent with what was found in earlier studies. This investigation highlighted the need to study pharmacogenetic relevance loci and documentation since evidence could be utilized to elucidate genetic backgrounds of drug metabolism, and provide a basis for future pharmacogenomic studies and adequate dose adjustments in Pakistani and global populations.

## 1. Introduction

Genetic constitution determines the varied interethnic metabolizing capacities of various drug metabolizing enzymes [1]. The recent developments in the pharmacogenomics field revealed that polymorphisms in DNA sequences identified as single nucleotide polymorphisms (SNPs) may elucidate some of the variability in drug metabolizing enzyme activities. These variations in DNA sequences contribute to inappropriate therapeutic responses in different ethnic groups. Geographical location and ethnicity are among the influential factors affecting the allelic distribution patterns of both phase-I and phase-II drug metabolizing enzymes [2].

Principally, the oxidative metabolism of xenobiotics, including most clinically used drugs, is mediated by the phase-I heme-containing members of the cytochrome P450 (CYPs) enzyme superfamily. Cytochrome P450 oxidoreductase (CYPOR) is indispensable in the metabolic activities mediated by all microsomal CYPs [3]. The CYPOR transfers two electrons from reduced nicotinamide adenine dinucleotide phosphate (NADPH) to microsomal enzymes for xenobiotics activation and ultimately convert them to highly active intermediate genotoxic metabolites [4]. These metabolites undergo subsequent detoxification by other drug metabolizing enzymes [2]. Mostly, phase-I drug metabolizing enzymes are highly polymorphic. However, the metabolic capabilities of these enzymes can range from completely lacking to ultrahigh activity, depending on its variation type. Examples of these types include copy number variations (CNVs), insertions and/or deletions (INDELs), and single nucleotide substitutions (SNSs) in protein-encoding genes. These changes in encoding regions can also result in an increased risk of adverse drug reactions, the interindividual variability in drug response, or the disease predisposition in different individuals [2]. Studies performed on different ethnicities and populations have demonstrated an extreme variation in the genotype and allele frequency distributions of *CYP2C9*, *CYP2D6*, and *CYPOR* genes. The basic phenotype or genotype findings have greatly contributed to the determination of an individual’s metabolic capacity that acts as an important tool for safe and rational drug administration. In the future, this practice will be beneficial to decrease the prevalence of adverse drug reactions and treatment failures particularly for narrow therapeutic index drugs (NTI-drugs) [2].

CYP2C9, one of the most abundant CYP enzymes, constitutes 50% of the CYP2C subfamily and is involved in the oxidative metabolism and clearance of up to 15–20% of clinically important drugs, including phenytoin and warfarin [5,6]. *CYP2C9* exists in different polymorphic forms and 60 alleles have been identified so far, most of which alter the metabolic activity of the enzyme [7]. CYP2C9*2 (rs1799853-R144C), CYP2C9*3 (rs1057910-I359L), and CYP2C9*14 (rs72558189-R125H) [8,9,10] are among the extensively studied defective alleles both in in vitro and clinical investigations [5,11]. These alleles are associated with reduced catalytic activity of the wild-type (CYP2C9*1). The CYP2C9*2 allele is more moderately defective while CYP2C9*14 was found to be highly defective in in vitro studies [11].

*CYP2D6*, “the only functional gene in the CYP2D human subfamily”, is one of the extensively investigated members in the genetic polymorphism context [12]. *CYP2D6* represents only 2% of total human hepatic CYPs and is non-inducible by environmental factors. This little amount is enough to metabolize more than 25% of currently marketed drugs. CYP2D6 substrate drugs including antidepressants, selective serotonin-reuptake inhibitors, antiemetics, beta-blockers, and opioids are mostly lipophilic in nature [13,14,15]. To date, more than one hundred *CYP2D6* alleles and sub-variants are available at the human CYP allele nomenclature website [16], with this number continuously increasing. Among the various *CYP2D6* reported alleles, some are fully functional, less functional and non-functional or null alleles [17]. A population of individuals can be considered as poor metabolizers (PMs), intermediate metabolizers (IMs), extensive metabolizers (EMs), and ultrarapid metabolizers (UMs) based on the individuals’ capabilities to deal with the CYP2D6 substrate [14]. Both rs16947 and rs1135840 SNPs are associated with ultrarapid metabolizer *CYP2D6* polymorphisms [18].

CYPOR is essential for all reactions catalyzed by CYPs for drugs, steroid hormones and xenobiotics [19]. All microsomal CYPs receive electrons from NADPH through CYPOR for their catalytic activities [20]. The expression of the CYPOR protein in the human liver is stoichiometrically lower than that of CYPs. The *CYPOR* gene is highly polymorphic and more than sixty SNPs have been identified relating to variations in CYPOR protein contents [21]. An online database listing all human *CYPOR* mutations and polymorphisms is available at [22]. The functional effects of various *CYPOR* allelic variants are CYP enzyme and substrate dependent [23,24].

By now, no investigation indicating the genotype and allele frequencies distributions of *CYP2C9*, *CYP2D6* and *CYPOR* genes in all Pakistani geographical populations is available. In the present work, we investigated the genotype and allele frequencies in *CYP2C9* (rs1799853, rs1057910 and rs72558189), *CYP2D6* (rs16947 and rs1135840) and *CYPOR* (rs1057868, rs781919285 and rs562750402) genes, and compared our findings with 11 corresponding world populations reported in the 1000 Genomes Project phase-III database [25]. Additionally, we attempted to employ in silico mutagenesis and three-dimensional protein structural alignment analysis approaches to explore the possible impacts of human *CYPOR* missense variations and to explain the influences of *CYP2C9* and *CYP2D6* SNPs corresponding to Pakistani people.

## 2. Materials and Methods

### 2.1. Study Participants, DNA Extraction and Quantification

In total, 244 (143 males and 101 females) healthy Pakistani volunteers from all provinces and administrative territories, aged 18–63 years, were randomly selected. The individuals were subdivided into smaller ethnic groups (KPK: Khyber Pakhtunkhwa; PUN: Punjab; SIN: Sindh; BAL: Balochistan; GLB: Gilgit-Baltistan; AZK: Azad Kashmir) based on reported ethnic classification [26] and the availability of volunteers living in China. For comparative analysis, we used 11 world populations data representing all continents (i.e., ASW: African ancestry in Southwest USA; LWK: Luhya in Webuye, Kenya; YRI: Yoruba in Ibadan, Nigeria; MEX: Mexican ancestry in Los Angeles, California, USA; CHB: Han Chinese in Beijing, China; CHS: Southern Han Chinese, China; JPT: Japanese in Tokyo, Japan; CEU: Utah, USA residents with Northern and Western European ancestry from the CEPH collection; TSI: Toscani in Italy; GIH: Gujarati Indians in Houston, Texas, USA; and STU: Sri Lankan Tamal in United Kingdom) retrieved from the 1000 Genomes Project phase-III database [75]. Individuals with a known history of liver disease and/or transplant, alcohol, or drug addiction and pregnant females were not included in this study. Demographic details of participants included are given in Table S1. The study purpose and experimental procedures were explained verbally and prior written informed consent was obtained from each participant. A small amount of peripheral blood (2 to 3 mL) was collected individually in ethylenediaminetetraacetic acid (EDTA) vacutainer tubes. Genomic DNA (gDNA) was extracted from whole blood samples using an AxyPrep Blood Genomic DNA Miniprep Kit (Axygen Biosciences 33210 Central Avenue, Union City, CA, USA) according to the manufacturer’s instructions. Extracted gDNA samples were quantified using a NanoDrop 2000 Spectrophotometer (Thermo Scientific, Wilmington, DE, USA) and integrity was then confirmed using agarose gel electrophoresis immediately after extraction. Dilutions of 50 ng/µL were prepared for each isolated DNA sample and stored at −40 °C until they were further used in polymerase chain reaction (PCR) analysis. The study was conducted in accordance with the Declaration of Helsinki, and the protocol was approved by the institutional ethical committee of Zhejiang University, College of Pharmaceutical Sciences (Hangzhou, China) with ethic approval number IRB-2017-2. To develop an understanding of how the Pakistani population differ in pharmacogenetic polymorphisms and frequency distributions of *CYP2C9*, *CYP2D6* and *CYPOR*, major allele variants from global populations and a comparative analysis technique was used. We compared our study results with world populations from all continents, as previously listed. The frequency distribution data of eleven world populations was retrieved from the 1000 Genomes Project phase-III database [25,27].

### 2.2. Polymerase Chain Reaction and Sequence Analysis

Individual gDNA was screened out by sequencing after PCR amplification for clinically relevant polymorphisms in *CYP2C9*, *CYP2D6*, and *CYPOR* genes. The targeted SNP sites were amplified using PCR, and Primer3 [28] was used to design all PCR primers (Supplementary Table S2). To avoid any polymorphic sites in the primer and to ensure the specificity of the primer sequence, National Center for Biotechnology Information database (NCBI) primers Basic Local Alignment Search Tool (BLAST) (https://blast.ncbi.nlm.nih.gov/Blast.cgi) and SNP BLAST (https://www.ncbi.nlm.nih.gov/snp) were used. Each 50 µL PCR reaction mix consisted of 1 µL of DNA (50 ng/µL), 5 µL 10× PCR buffer, 2 µL of each primer (1000 µM), 4 µL deoxynucleotide triphosphate (2.5 mM), and 0.325 µL r-Taq polymerase (5 U/µL). Amplification was performed using an Eppendorf (AG 22331 Hamburg, Germany) thermal cycler, and the PCR profile included an initial denaturation of 2 min at 94 °C followed by 32–34 cycles of 30 S at 94 °C, 20 S at 60 °C, and 30 S at 72 °C; a final extension step at 72 °C for 10 min; and being held at 4 °C. Amplicons were confirmed for band sufficiency and specificity on a transilluminator following agarose gel electrophoresis, and they were purified before sequence analysis. Purified amplicons were sequenced using the same forward and reverse primers by Sangon Biotech Co., Ltd. (Shanghai, China). For the detection of polymorphisms in *CYP2C9*, *CYP2D6* and *CYPOR* genes, all SNPs were identified followed by human reference genome assembly (GRCh38). The full length standard reference sequences for human *CYP2C9* (NC_000010.11), *CYP2D6* (NC_000022.11) and *CYPOR* (NC_000007.14) genes were obtained from the GenBank, NCBI [29] and used BLAST for nucleotide and each fragment sequence read was aligned to determine the particular chromosomal variation location of SNPs (NCBI, Bethesda, MD, USA) [30,31] and SNPs were verified from NCBI Variation Resources [32], the single nucleotide polymorphism database (dbSNP), and the Exome Sequencing Project (ESP) (http://evs.gs.washington.edu/EVS/). For accuracy, every DNA fragment was sequenced in both the forward and reverse directions, and sequence reads were observed for base pair changes to identify the specific variations using Chromas Lite software, (version 2.6.5, Technelysium Pty. Ltd., Tewantin, Queensland, Australia) [33]. The counting method was used to calculate allele and genotype frequencies, coupled with manual inspection of the sequence chromatograms, accuracy was ensured by at least two distinct individuals.

### 2.3. Statistical Analysis

For each polymorphism, the chi-square test was applied to analyze the genotype frequencies for Hardy–Weinberg equilibrium using an electronic calculator [34]. To determine the global allele frequency differences between various racial groups, pairwise comparisons among pairs of populations available in 1000 Genomes Project Phase-III database and Pakistani population were carried out, and the *p* < 0.05 level was considered as statistically significant. Intraethnic allele frequency comparison between different Pakistani regions was carried out using one-sided binomial proportion test. For each test, calculated *p*-values were adjusted for the multiple testing using q-value (Supplementary Table S3). The freely available statistical package R [35] software was used for all statistical analyses. The basic characteristics of all studied polymorphism genotypes are summarized in (Table 1).

### 2.4. In Silico Mutagenesis Analysis: Modeling of CYP2C9, CYP2D6, and CYPOR Variant Protein Structures

The X-ray structures of CYP2C9 (PDB ID: 1OG2), CYP2D6 (PDB ID: 2F9Q), and CYPOR (PDB ID: 3QE2) were retrieved from the worldwide available archive for structural data of biological macromolecules, the Protein Data Bank (PDB) [36]. They were used as starting templates for the construction of CYP2C9, CYP2D6, and CYPOR variant models using in silico mutagenesis corresponding to the Pakistani population. Missense variations in *CYP2C9*, *CYP2D6* and *CYPOR* genes were confirmed in the Pakistani population by successful sequence analysis and verified from the single nucleotide polymorphism database (dbSNP) of NCBI. In silico mutagenesis of identified point substations were created in the 3D protein structures of wild-type CYP2C9, CYP2D6 and CYPOR after deleting water molecules. For the evaluation of the structural/functional effect of every SNP, each amino acid replacement corresponding to a reported missense variation was made individually. The process was performed by keeping the replaced residues free, fixing the rest of structure, taking into account the secondary structure protein features, and independent ultimate structural aligning to the corresponding wild-type. The modeling procedures were performed using SYBYL 6.0 software (Tripos 1699, St Louis, MO, USA) and the molecular visualizations were performed on PyMOL Molecular Graphics System, Version 1.6 (Schrödinger, LLC) [37,38,39].

## 3. Results and Discussion

**Figure 1:**
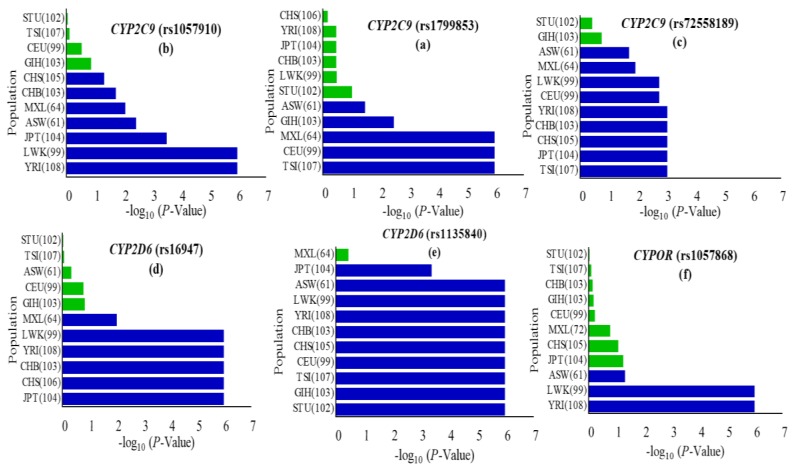
Occurrence of cytochrome P450 2C9 (*CYP2C9*), cytochrome P450 2D6 (*CYP2D6*) and cytochrome P450 oxidoreductase (*CYPOR*) missense variants in the Pakistani cohort vs. eleven world populations represented in the 1000 Genomes Project. The *p*-value of the χ2 test in *CYP2C9*, *CYP2D6* and *CYPOR* missense variants among the Pakistani cohort vs. global populations were calculated using the chi-square test and values were considered significant at the *p* < 0.05 level. Statistically significant variations are represented by a blue bar (**a**–**f**). Global populations are arranged according to the least significant to most significant negative log(*p*-value). Populations included were African ancestry in Southwest USA (ASW); Luhya in Webuye, Kenya (LWK); Yoruba in Ibadan, Nigeria (YRI); Mexican ancestry in Los Angeles, CA, USA (MEX); Han Chinese in Beijing, China (CHB); Southern Han Chinese, China (CHS); Japanese in Tokyo, Japan (JPT); Utah, USA residents with Northern and Western European ancestry from the Centre d’Etude du Polymorphisme Humain (CEPH) collection (CEU); Toscani in Italy (TSI); Gujarati Indians in Houston, Texas, USA (GIH); Sri Lankan Tamal in UK (STU). Numbers in parentheses indicate the group size.

In the recent era of genomics, due to new advances in technologies, a direct DNA sequencing approach can be easily applied to ascertain the genotype of an individual and to identify any change in human DNA sequences. Globally, this approach has already been practiced in various human genes not only to determine numerous polymorphisms but also to identify individual’s SNPs associations to disease phenotypes [40,41]. Genetic variations in phase-I and phase-II drug metabolizing genes have long been as substantial elements related to inconstant drugs and xenobiotics metabolisms. Therefore, genotype and allele distribution based investigations of these genes in the Pakistani population are of absolute importance as the Pakistani population has become a heterogeneous admixture of genetic diversity due to the invasions of the British and Arabs colonialism [42] and is now the second most populated South Asian country. Out of 244 Pakistani individuals living in China representing all provinces and administrative territories, whole genome sequence analysis of selected individuals (one from each province, unpublished data) showed the highest number of variations in *CYP2C9*, *CYP2D6*, and *CYPOR* compared to others. All DNA samples extracted from healthy volunteers’ venous blood were analyzed for thirty-six SNPs in coding and related noncoding regions (Table 1) of these genes. Regional business trends of the Pakistani population and living in China influenced the varied availability of healthy individuals and, hence, the size of some groups. Exclusion of some bad quality sequencing chromatograms resulted in a variable number of tested samples. For all SNPs included in the present investigation, calculated genotype and allele frequencies in the Pakistani population were compared to global populations (African, Admixed American, East Asian, European, South Asian, etc.) provided in the 1000 Genomes Project Database Phase-III database [25] (Supplementary Table S3). The population-based comparative studies of genetic variants or SNPs in drug metabolizing genes are of immense worth, because genetic differences are heavily related to ethnicity [43]. Genotype distributions of identified polymorphisms were calculated to determine whether there was Hardy–Weinberg equilibrium (*p* < 0.05) (Supplementary Table S4). The distribution of allele and genotype frequencies showed a significant dissimilarity to most of the compared global ethnic groups (Figure 1 and Supplementary Table S3).

### 3.1. CYP2C9 Polymorphisms

**Figure 2:**
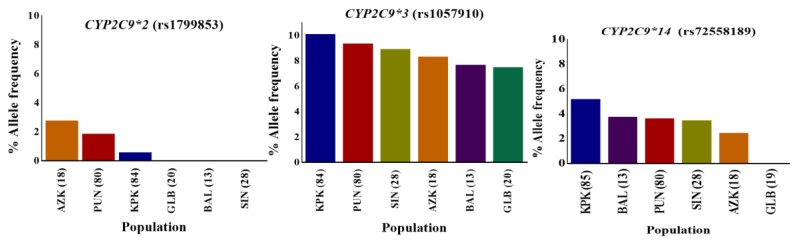
CYP2C9*2, CYP2C9*3, and CYP2C9*14 variant allele distributions in indigenous Pakistani populations. The distribution of the percentage allele frequency of pharmacogenetic CYP2C9*2, CYP2C9*3 and CYP2C9*14 variants in Pakistani local populations. Regional populations are represented by colored columns in decreasing variant allele percentage frequency order. The CYP2C9*2, CYP2C9*3, and CYP2C9*14 variants are associated with reduced enzyme metabolic activity in comparison to the CYP2C9*1 wild-type. The CYP2C9*2 variant was found most frequently in the AZK region while CYP2C9*3 and CYP2C9*14 allele variants were in relatively higher frequencies in individuals from KPK. However, these variants were least frequent in GLB territory, as well as CYP2C9*2. KPK: Khyber Pakhtunkhwa; PUN: Punjab; SIN: Sindh; BAL: Balochistan; GLB: Gilgit-Baltistan; and AZK: Azad Kashmir. Numbers in parentheses indicate the group size.

**Figure 3:**
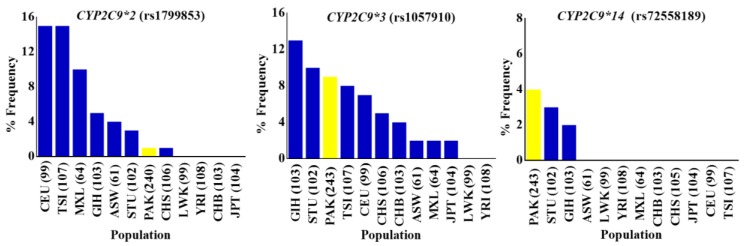
The allele frequency distribution of CYP2C9*2, CYP2C9*3, and CYP2C9*14 variants in indigenous Pakistani and global populations. Percentage allele frequency distributions of CYP2C9*2 (rs1799853), CYP2C9*3 (rs1057910) and CYP2C9*14 (rs72558189) pharmacogenetic variants in the Pakistani population and global populations from the 1000 Genome Project depicted in bar chart plots. Frequencies in the Pakistani population are shown in yellow, while others are represented by blue colored columns in the order of decreasing variant allele percentage frequency. The CYP2C9*2, CYP2C9*3, and CYP2C9*14 three *CYP2C9* missense variants associated with reduced enzyme activities, were found to be almost the least frequent, intermediately frequent, and highly frequent respectively, in indigenous Pakistani individuals compared with global populations. PAK: Pakistani, Pakistan; ASW: African ancestry in Southwest USA; LWK: Luhya in Webuye, Kenya; YRI: Yoruba in Ibadan, Nigeria; MXL: Mexican ancestry in Los Angeles, California, USA; CHB: Han Chinese in Beijing, China; CHS: Southern Han Chinese, China; JPT: Japanese in Tokyo, Japan; CEU: Utah, USA residents with Northern and Western European ancestry from the CEPH collection; TSI: Toscani in Italy; GIH: Gujarati Indians in Houston, Texas, USA; STU: Sri Lankan Tamal in UK. Numbers in parentheses indicate the group size.

*CYP2C9*, one of the most abundant members of the human *CYP2* isoform, has been extensively studied for polymorphisms in different ethnicities including Caucasian, African-American, and Asian populations [44,45,46]. CYP2C9 is involved in the metabolism of clinically important drugs, including most of the nonsteroidal anti inflammatory drugs (NSAIDs) and others like phenytoin, warfarin, and prasugrel. The United States Food and Drug Administration (FDA) approved phenytoin, warfarin and prasugrel among the drugs with pharmacogenomic information in their labeling (www.fda.gov/default.htm). The *CYP2C9* gene was successfully genotyped for rs1799853 (CYP2C9*2/R144C), rs1799853 (CYP2C9*2/R144C), and rs72558189 (CYP2C9*14/R125H) polymorphisms, and allele frequencies were calculated for them. The frequencies of rs1799853, rs1057910 and rs72558189 variant alleles were calculated as 1.04, 9.26, and 4.12%, respectively. Genotype frequencies of rs1799853 and rs1057910 did not deviate significantly from Hardy–Weinberg equilibrium, while the *p*-value for rs72558189 was calculated as <0.05 (Supplementary Table S4). The distribution comparisons of these *CYP2C9* variants within Pakistani populations and with global populations have shown the existence significant intra- and inter-population differences, respectively (Figure 2 and Figure 3). We found that the genotype and variant allele distribution of rs1799853 in the Pakistani population is similar to that of other South Asians subjects but about 15 times less frequent than in European populations. In the case of a rs1057910 polymorphism, the variant allele occurred with a frequency of 9.62% in Pakistani subjects, which is significantly higher than in African, Admixed American, and East Asian populations (*p* < 0.005), as shown in Figure 1 and Supplementary Table S3. These findings are consistent with previously reported 1000 Genomes Project data for South Asian and European populations, in which the frequency of the rs1057910 variant allele were 9.80, 13.1 and 8.40, 6.60%, respectively, as shown in Supplementary Table S3. The *CYP2C9* polymorphisms rs1799853 (CYP2C9*2), rs1057910 (CYP2C9*3), and rs72558189 (CYP2C9*14) are variants associated with reduced enzymatic activity leading to poor metabolizing phenotype [9,10,11]. In relation to wild-type, rs1799853 and rs72558189 polymorphisms, respectively, demonstrated 16–20 and 4–6% enzymatic activity [8]. An in vitro study revealed that rs72558189 polymorphism results in a highly defective CYP2C9 protein that leads to more than 90% reduction in tolbutamide clearance [11]. The occurrence of this exome variant in Pakistani subjects was found higher than any other population included in the present study. The frequency of the variant allele is 4.12% in the Pakistani population while this allele was not reported in African, American, East Asian, and European individuals (Supplementary Table S3). Until now, no study was available, in which this highly defective variant allele is described in the Pakistani population. The present study indicated a high prevalence of CYP2C9*14 defective allele in the Pakistani population and will provide a clear indication for genetic test benefits in narrow therapeutic range substrate dosing, such as for warfarin and phenytoin (Supplementary Table S4 and Figure S3). This will also help physicians to manage the effective dosage for other CYP2C9 substrates.

### 3.2. CYP2D6 Polymorphisms

The highly polymorphic *CYP2D6* is one of the most studied genes in the cytochrome P450 family for ethnic distributions and allelic variations. Allelic distribution patterns of *CYP2D6* are influenced by geographical location and ethnicity, while genetic composition determines the variability of interethnic drug metabolizing capacities of concerned proteins [1,47]. Polymorphisms rs16947 and rs1135840 were genotyped and analyzed for their frequencies in the *CYP2D6* gene for 241 and 240 Pakistani individuals, respectively. Related to these exome variants, 13 SNPs in noncoding regions were also genotyped. The variant allele A frequency of rs16947 polymorphism was observed 38.17% in individuals and calculated in comparatively higher (52.78%) percentages in individuals from the Azad Kashmir (AZK) region. Polymorphism rs1135840 related variant allele G was calculated as having a 35.21% frequency and observed to be significantly different from Hardy–Weinberg equilibrium with a *p*-value of <0.05, as shown in Supplementary Table S4. The genotype distribution of rs16947 in the Pakistani population significantly differed from East Asians, Admixed Americans, most Africans, and Europeans but was similar to the other South Asians, while rs1135840 polymorphism differed from all other populations (*p* < 0.05), shown in Figure 1 and Supplementary Table S3. In the present study, both identified rs16947and rs1135840 polymorphisms are responsible for *CYP2D6* gene products with the ultrarapid metabolizer phenotype [48,49]. A recent study by Zeng et al. revealed that carriers of variant alleles A and G of rs16947 and rs1135840 polymorphisms, respectively, require higher risperidone doses due to faster elimination rates of CYP2D6 to improve cognitive performance in patients with schizophrenia [49]. The SNP rs1135840 was found not to be associated with decreased CYP2D6 enzyme activity for bufuralol or dextromethorphan in an in vitro investigation when compared with the normal CYP2D6 enzyme [50]. Zhou et al. reported the rs1135840 heterozygote (GC) as a cancer-associated potential risk factor in carriers and a direct association between cancer risk and heterozygosity (GC) was found [51]. The rs1135840 heterozygote (GC) frequency was 69.58% in the present investigation, which is significantly higher than all the other compared populations, which may result in a different metabolic phenotype. The homozygote (CC) of rs1135840 in the *CYP2D6* gene, suggested to be a protective factor for cancer risk, however, was found less frequently (30%) in the Pakistani population. The homozygote (GG) and G carriers (AG) of rs16947 polymorphism in the *CYP2D6* gene were reported to be negatively associated with cancer incidences and considered to be protective against cancer risks [51]. The cancer protective genotypes AG and GG were found most frequently (48.96 and 37.35%) in the current study, with the highest (68.86%) G allele frequency found in Sindhi ethnic individuals similar to other South Asian populations (Supplementary Tables S3 and S4). The rs16947 variant genotype was stated to be associated with lower complete remission rates of acute myeloid leukemia compared to patients with the wild genotype [52], whereas the rs16947 variant allele also appeared to be associated with drug induced bradycardia in open angle glaucoma individuals, while allele G of rs1135840 found no such association compared to other alleles [53].

### 3.3. CYPOR Polymorphisms

**Figure 4:**
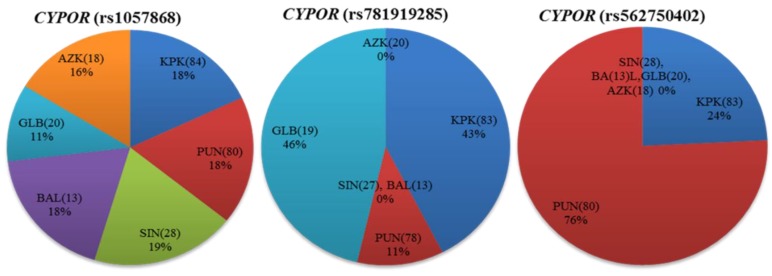
Distribution of the cytochrome P450 oxidoreductase (*CYPOR*) missense variants among Pakistani indigenous populations. Percentage allele frequency distributions of *CYPOR* (rs1057868, rs781919285 and rs562750402) are shown as pie charts for different regions of Pakistan. Numbers in parentheses indicate the group size.

The *CYPOR* gene product is an important electron donating flavoprotein and essentially required for all reactions catalyzed by CYPs. The *CYPOR* gene was successfully genotyped for three nonsynonymous (i.e., rs1057868, rs781919285, and rs562750402), four synonymous (i.e., rs1057870, rs2228104, rs369026313, and rs1135612) substitutions, and 11 related noncoding variants. The *CYPOR* rs781919285 and rs562750402 human variations were reported only in 0.001652 and 0.002480%, respectively, in dbSNP, and no information was provided in the Human Cytochrome P450 (CYP) Allele Nomenclature Database [22] and the 1000 Genomes Project phase-III database [25]. For the first time in the present study, both are identified relatively in higher interethnic (1.25 and 1.65%) frequency distributions. Based on intraethnic distributions, rs781919285 was dominantly distributed (2.63%) in Gilgit–Baltistan (GLB), while rs562750402 was 3.75% in Punjab (PUN) regions (Supplementary Table S4). In the *CYPOR* gene, the non-synonymous rs1057868 polymorphism was statistically significant in African and Japanese ethnicities, while rs1057870 and rs2228104 synonymous variations were found significantly different from African, East Asian, and Admixed American population groups. The rs1135612 polymorphism identified in the *CYPOR* gene was observed statistically significant in genotype and allele frequency distributions in the studied population, when compared with previously reported populations (*p* < 0.05) shown in Supplementary Table S3. The two *CYPOR* rs781919285 and rs562750402 human variations were not reported in any of the compared populations. However, in the Pakistani population, though there were relatively diverse intraethnic frequencies, *CYPOR* rs1057868 was found to be almost uniformly distributed (Figure 4). Polymorphisms in the *CYPOR* gene have been found to be associated with different disease conditions like the Antley–Bixler syndrome, amenorrhea, congenital adrenal hyperplasia and steroidogenesis disorders [54,55,56]. The allele frequency of the rs1057868 variant was found as 32.51% in the Pakistani population. Agrawal et al. observed a 67% reduced CYP3A4 activity from rs1057868 polymorphism [57]. Patients with rs1057868 homozygous (TT) genotype exhibited an increased metabolic clearance of midazolam and nicotine compared to subjects with a normal CC genotype [58,59]. Kidney transplant patients with mutant allele T required high doses of immunosuppressant drugs, like tacrolimus and rapamycin, and are at increased risk of developing diabetes mellitus compared to ancestral allele C carriers [60,61], whereas, in children with familial hypercholesterolemia, both homozygous (TT) and heterozygous (CT) variant genotypes required lower doses of cholesterol-lowering agents like atorvastatin, compared to CC genotype subjects. The TT genotype was, however, reported to be linked with lower bladder cancer incidences in the Chinese population [62]. The distribution of rs1057868 variant allele T in the studied subjects is calculated higher than Africans and Mexican Americans, while the finding is consistent with other South Asian populations (Supplementary Table S3).

### 3.4. In Silico Influences of SNPs on the Structure and Functions of CYP2C9, CYP2D6, and CYPOR Proteins

**Figure 5:**
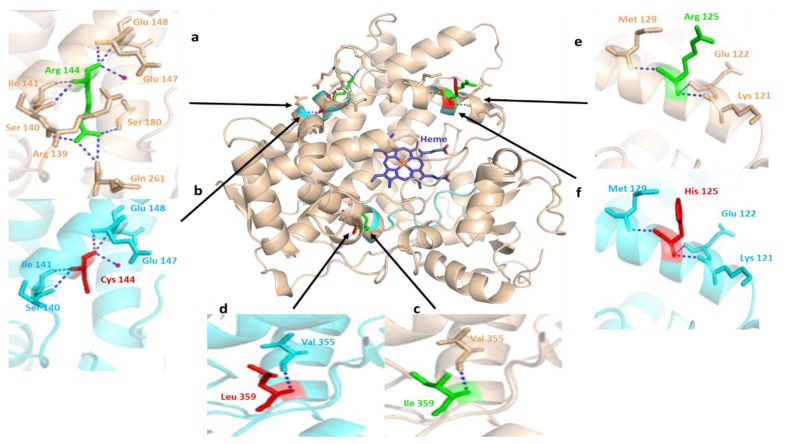
The structural alignment of human CYP2C9 (PDB: 1OG2) to the substrate free mutant CYP2C9 protein obtained by in silico mutagenesis. Hydrogen bonds are represented as a blue dotted line and the water molecule as hot pink spheres. The heme domain is shown in blue, while arrows indicate the deviation sites among the structures of normal (green) and mutated (red) proteins found in the Pakistani population. The wheat colored a, c, and e, and cyan b, d, and f show the 3D representations of normal and altered hydrogen bond interactions corresponding to amino acid changes, respectively. (**a**) a close view of arginine amino acid at the position of 144 intermolecular interactions. The residue has an intensive hydrogen bond network with the R139, S140, I141, E147, E148, S180 and Q261 backbone polypeptide amino acids and a tightly bounded water molecule; (**b**) visualization of hydrogen bond networking perturbation in the CYP2C9 crystal structure, produced by in silico mutagenesis of Arg144 with a relatively small 144Cys residue. This in silico mutagenesis resulted in the breaking of three intermolecular hydrogen interactions, with the adjacent arginine 139, glutamine 261, and serine 180 leading to altered CYP2C9 protein interaction with CYPOR [9]; (**c**) intramolecular hydrogen bonding of wild-type Ile359 located in the substrate recognition site. It is important in substrate specificity and affinity determination [75]; (**d**) in silico produced missense mutation visualization in the CYP2C9 crystal structure by the substitution of Ile359 with Lue 359; (**e**) Arg125 lies near the heme domain, on the solvent-exposed protein surface, and interacts with NADPH-P450 reductase in NADPH-dependent metabolisms [75]; (**f**) a partial view of the missense mutation resulting in the replacement of Arg125 with 125His. This leads to varied interactions necessary for NADPH-dependent CYP2C9 metabolic activity [76].

Different structure-based in silico studies have been effectively used as complementary approaches to investigate the effects of missense human variations on the drug metabolizing capacities of CYPs and the functions of other gene products due to genetic polymorphisms [63,64,65,66,67]. The successful structural alignment of human CYP2C9 (PDB: 1OG2) to the substrate-free mutant CYP2C9 protein model obtained by in silico mutagenesis revealed varied binding modes of normal and substituted protein point variations identified in the Pakistani population (Figure 5). The amino acid Arg144 is located on the protein surface away from the enzyme active site. A previously known SNP (rs72558189) resulted in R144C substitution leading to a defective (CYP2C9*2) variant allele [68]. The present in silico mutagenesis study has depicted that the replacement of Arg144 with relatively smaller Cys144 residue lead to the destabilization and perturbation of three intermolecular hydrogen interactions, with adjacent Arg139, Glu261 and Ser180 (Figure 5a,b) residues. The resulting change in intermolecular electrostatic interactions may be involved in the reduced activity of CYP2C9 due to the altered interaction between CYPOR and CYP2C9. The findings of the present investigation are consistent with previous studies [9,69]. Amino acid isoleucine at the 359 position directly contributes to CYP2C9 substrate recognition due to its unique location in the CYP2C9 protein active site [64]. The substitution of Ile359 with other residues indirectly infers CYP2C9 metabolic activity. Figure 5c,d are the close visualizations of the wild-type and mutant-type CYP2C9 crystal structure, respectively, generated by in silico mutagenesis. No change in the structure or in the electrostatic interaction of Ile359Leu to the peptide backbone was observed, but a larger space on top of the binding pocket was suggested in another computational study [69]. Therefore, the amino acid replacement Ile359Leu due to the space expansion over the binding pocket will be unable to confine the substrate to the heme center vicinity. This is unfavorable compared to CYP2C9 wild-type metabolic activity, leading to a markedly defective CYP2C9*3 allele [70]. The present three-dimensional structural alignment of CYP2C9 revealed that the arginine residue at position 125 is located near the heme domain on the protein surface. The amino acid Arg125 forms hydrogen interactions with Lys121, Glu122, and Met129 residues in the peptide backbone (Figure 5e). Arg125 is highly conserved in the CYP2 family and is equivalent to Arg126 in CYP2B6. A previous study showed that Arg126 is one of the Cyt b5 and NADPH-P450 reductase binding site residues [71]. The in silico mutagenesis of Arg125 with relatively shorter side chain histidine residue has no effect on hydrogen bond interactions with the peptide backbone amino acids (Figure 5f) but lead to decreased electrostatic interactions with CYPOR [64]. This decreased electrostatic interactions resulted in a highly defective CYP2C9*14 allele [8,11].

**Figure 6:**
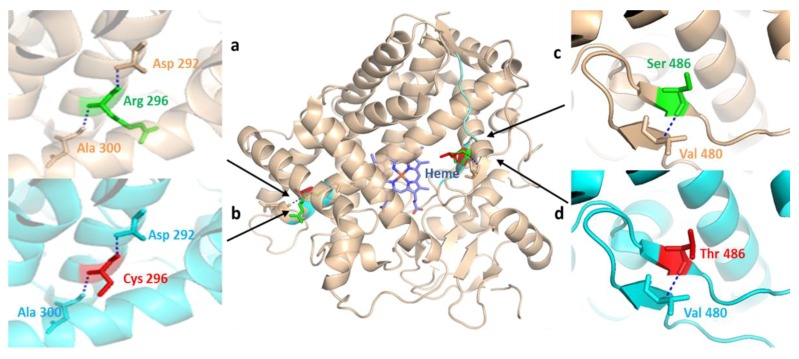
Structural comparison of human CYP2D6 (PDB: 2F9Q) to the mutant CYP2D6 protein depicting normal and missense mutation sites reported in the Pakistani population. Wild-type CYP2D6 is represented in wheat, while mutant-type is represented in cyan. Positions of normal (green) and mutant (red) amino acid residues are indicated by black arrow heads. (**a**) the hydrogen interactions of wild-type Arg296 residue to peptide backbone Asp292 and Ala300 residues; (**b**) X-ray structure partial view of missense SNP produced by in silico mutagenesis through the substitution of positively charged polar Arg296 with the relatively smaller nonpolar Cys296 residue; (**c**,**d**) highlight hydrogen bond interactions of wild-type Ser486 and mutant Thr486 amino acids with the adjacent Val480 residue in peptide backbone, represented in wheat and cyan, respectively.

Also examined were the structural differences from aligning the 3D structures of CYP2D6 (PDB: 2F9Q) wild type and two site mutants generated by in silico mutagenesis found in Pakistani people (Figure 6). The amino acid Arg296 lies near one of the substrate recognition sites away from the heme iron [72]. The outward direction of the residue Arg296 may hinder in its direct interaction with the substrate and interact with Asp292 and Ala300 amino acids through hydrogen bonds (Figure 6a). The substitution of positively charged polar Arg296 with the relatively smaller nonpolar Cys296 residue is a common *CYP2D6* gene variation and reported in combination with others in several *CYP2D6* alleles [16]. The substitution of Arg296 with Cys296 introduced by in silico mutagenesis has not altered the hydrogen interactions with the peptide backbone (Figure 6b), but a highly decreased catalytic efficacy associated with this change was previously observed [73]. The other missense variation detected in the Pakistani population resulted in the substitution of Ser486 with Thr486 in the *CYP2D6* gene. The Ser486 residue is located distal to the protein active site but is nearer to the solvent channel [72]. The structural alignment study of S486T substitution showed no obvious structural changes and alterations in hydrogen bond interactions with the peptide backbone (Figure 6c,d). The missense SNP S486T is frequently reported in other *CYP2D6* allele variants [16] and is associated with diverse catalytic activities [74].

**Figure 7:**
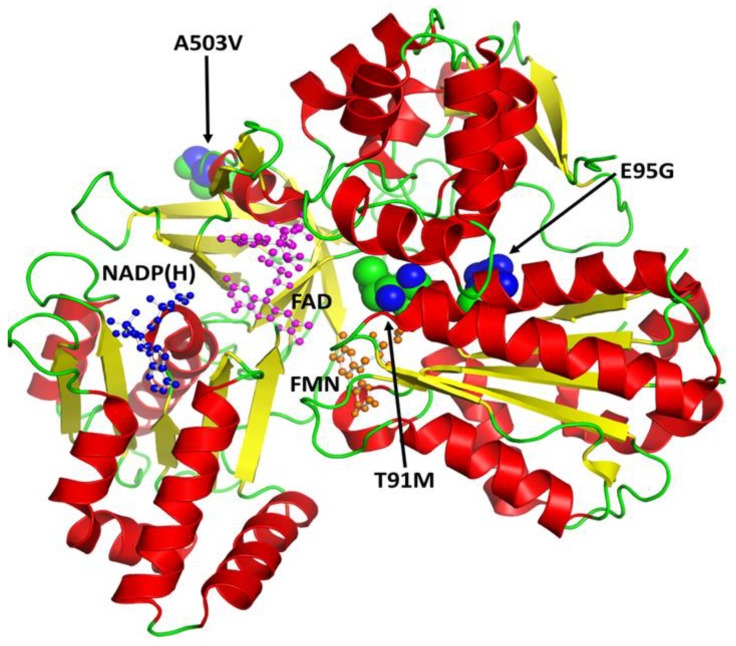
A secondary structural feature-based (helices in red, sheets in yellow and loops in green) schematic illustration of the human cytochrome P450 oxidoreductase (CYPOR). The co-factors FAD (magenta), NADP(H) (blue), and FMN (orange) are shown as ball and stick models. Missense mutations identified in the Pakistani population are shown as spheres.

**Figure 8:**
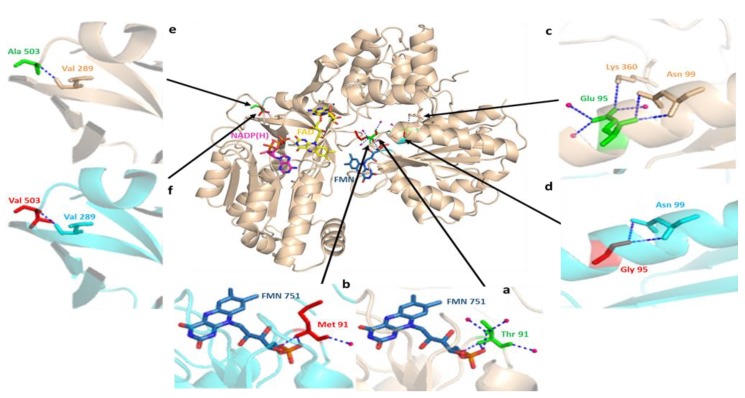
Three-dimensional structural alignment of wild-type human CYPOR (PDB: 3QE2) and mutant protein produced by in silico mutagenesis, showing missense mutations corresponding to the Pakistani population (wheat color) and wild-type (cyan color) peptide backbones. Cartoon structures of human CYPOR are depicted, as well as cofactors FMN (blue-gray) and FAD (yellow) and coenzyme NADP(H) (pink) in stick configuration. The interaction analysis of the wild-type and mutant residues strongly suggest that the two structures of human CYPOR are almost identical, except for the sites of missense SNPs. Partial views of crystal structures show a hydrogen bonding network involving water molecules (hot pink) and peptide backbones (blue dotted lines). (**a**) the wild-type Thr91 residue shows three hydrogen interactions each with the adjacent flavin mononucleotide (FMN) cofactor and water molecules; (**b**) the substitution of polar Thr91 with the nonpolar Met91 residue resulted in the loss of three hydrogen interactions, one with FMN and two with water molecules that were involved in its stabilization; (**c**) wild-type Glu95 directly interact with Asn99, Lys360 residues, and water molecules via hydrogen bonding; (**d**) a change in the hydrogen bonding network is observed due to the replacement of the negatively charged Glu95 with the nonpolar Gly95 residue; (**e**) hydrogen interaction between wild-type Ala503 and adjacent nonpolar Val289 residue; (**f**) apparently, the replacement of wild type Ala503 residue with Val503 does not affect the bonding interactions with the peptide backbone.

The identified missense variations in the Pakistani population are shown in a typical three-dimensional structural of human CYPOR protein molecule in Figure 7. A three-dimensional structural alignment of normal and mutant CYPOR models (Figure 8) highlights the effect of point variations in protein structures. The interaction analysis of the wild-type and substituted residues with others, revealed that the models of human CYPOR are structurally conserved, except the sites of missense SNPs. Partial views of crystal structures exposed a hydrogen-bonding network involving water molecules (hot pink) and peptide backbones (blue dotted lines). The Thr91 residue, being part of flavin mononucleotide (FMN) binding pocket, shows six hydrogen interactions, three each with the FMN cofactor and water molecules involved in its stabilization (Figure 8a) [19]. In silico mutagenesis of the polar Thr91 residue with the larger nonpolar Met91 resulted in the loss of three hydrogen interactions, one with FMN and two with water molecules (Figure 8b). The polar Glu95 amino acid, with a negatively charged side chain, is a part of the FMN domain (70 to 208) [39]. The Glu95 residue directly interact with Asn99, Lys360 residues, and water molecules via hydrogen-bonds (Figure 8c). A disruption of four hydrogen interactions was observed due to the replacement of Glu95 with the nonpolar Gly95 residue, the Lys360 residue (a part of the CYPOR flexible hinge region), and three water molecules (Figure 8d). The nonsynonymous variations, Glu95Gly and Thr91Met, in the CYPOR enzyme will modify the FMN binding pocket configuration due to altered electrostatic attractions. This, in turn, will change the electron transfer to redox partners, like CYPs and their metabolic capabilities. The residue Ala503 is the part of the flavin adenine dinucleotide (FAD) binding domain and directly interacts with Val289 in the peptide backbone through a hydrogen bond (Figure 8e). The substitution of Ala503 with Val503 did not disturb the peptide backbone interactions (Figure 8f), but this change led to the disruption of NADPH interactions, which resulted in moderately reduced cytochrome c reduction and P450C17 hydroxylation [77,78]. The diverse influence of Ala503Val substitution has recently been observed in drug and xenobiotic metabolism investigations [57,79].

## 4. Conclusions

To the best of our knowledge, this study is the first to reveal the identification of rare *CYPOR* SNPs (rs781919285 and rs562750402) following relatively diverse intraethnic, interethnic and distribution patterns and the detection of defective *CYP2C9* (rs72558189) and *CYP2D6* (rs1135840) polymorphisms with the highest frequencies in the healthy Pakistani population. This study also supports how allele frequency distributions of human biotransformation genes follow geographic and/or diverse ethnic specific patterns rather than random distributions.

## Figures and Tables

**Table 1:** Basic characteristics of studied genotyped polymorphisms.

Gene.	SNP ID	Amino Acid Substitution	^a^ Chromosomal Position	^b^ SNP Function	^c^ MAF (Pak)
CYP2C9	rs1799853	R144C	chr10:94942290	missense	0.0479 (0.0104)
CYP2C9	rs1057910	I359L	chr10:94981296	missense	0.0485 (0.0926
CYP2C9	rs72558189	R125H	chr10:94942234	missense	0.0042 (0.0412)
CYP2D6	rs16947	R296C	chr22:42127941	missense	0.3592 (0.3817)
CYP2D6	rs1135840	S486T	chr22:42126611	missense	0.4012 (0.3521)
CYP2D6	rs566383351		chr22:42131222	intron variant	0.0004 (0.1459)
CYP2D6	rs530422334		chr22:42131145	intron variant	0.0002 (0.0207)
CYP2D6	rs35023634		chr22:42131023	intron variant	0.0004 (0.0014)
CYP2D6	rs35481113		chr22:42131016	intron variant	0.0006 (0.0166)
CYP2D6	rs543939200		chr22:42130994	intron variant	0.0002 (0)
CYP2D6	rs372204775		chr22:42130993	intron variant	0.0084 (0.09)
CYP2D6	rs267608272		chr22:42130929	intron variant	0.0022 (0)
CYP2D6	rs28371725		chr22:42127803	intron variant	0.0635 (0.1958)
CYP2D6	rs28371738		chr22:42126390	intron variant	0.2368 (0.1033)
CYP2D6	rs374672076		chr22:42131189	intron variant	NA (0.127)
CYP2D6	rs17002852	H232H	chr22:42128321	synonymous	0.0072 (0.0271)
CYP2D6	rs34167214		chr22:42131122	intron variant	NA (0.5)
CYP2D6	rs35046171		chr22:42131063	intron variant	NA (0.1339)
CYPOR	rs1057868	A503V	chr7:75985688	missense	0.2861 (0.3251)
CYPOR	rs1057870	S572S	chr7:75985969	synonymous	0.1953 (0.2851)
CYPOR	rs781919285	E95G	chr7:75979497	missense	0.000009 (0.0042)
CYPOR	rs562750402	T91M	chr7:75979485	missense	0.00003 (0.0062)
CYPOR	rs71526806		chr7:75985545	intron variant	NA (0.095)
CYPOR	rs4732514		chr7:75984680	intron variant	0.4952 (0.5729)
CYPOR	rs4732515		chr7:75984711	intron variant	0.1881 (0.0612)
CYPOR	rs4732516		chr7:75984764	intron variant	0.1881 (0.0599)
CYPOR	rs2228104	A485A	chr7:75985635	synonymous	0.1947 (0.0702)
CYPOR	rs2302433		chr7:75985882	intron variant	0.0246 (0.0166)
CYPOR	rs1135612	P129P	chr7:75980359	synonymous	0.2494 (0.2303)
CYPOR	rs13223707		chr7:75983452	intron variant	0.1887 (0.0622)
CYPOR	rs13240147		chr7:75983465	intron variant	0.1889 (0.1889)
CYPOR	rs41301394		chr7:75983485	intron variant	0.275 (0.3229)
CYPOR	rs10239977		chr7:75979668	intron variant	0.1963 (0.2063)
CYPOR	rs533975609		chr7:75979781	intron variant	0.0016 (0.0232)
CYPOR	rs72554000		chr7:75979826	intron variant	0.0106 (0.0167)
CYPOR	rs369026313	D163D	chr7:75980461	synonymous	0.00003 (0)

^a^: According to the human reference genome assembly (GRCh38), UCSC Human Genome Browser; ^b^: Based on the single nucleotide polymorphism database (dbSNP); ^c^: Indicates the frequency of the minor allele (MAF), according to dbSNP (Pakistani population); single nucleotide polymorphism (SNP).

## Supplementary Materials

The following are available online at http://www.mdpi.com/2073-4425/9/10/514/s1, Table S1: Demographics of the subjects included in present study, Table S2: Primers used for CYP2C9, CYP2D6 and CYPOR SNPs amplification and sequencing, Table S3: Distribution of Cyp2C9, Cyp2D6 and CYPOR variants in Pakistani population and global populations from 1000 Genomes Project Database, Table S4: Genotype and Allele (%) frequency distributions of CYP2C9, CYP2D6and CYPOR SNPs in indigenous Pakistani populations.
